# History and Perspectives of Nuclear Medicine in Myanmar

**DOI:** 10.22038/aojnmb.2017.9905

**Published:** 2018

**Authors:** Win Mar

**Affiliations:** Department of Nuclear Medicine, Yangon General Hospital, Yangon, Myanmar

## Abstract

Nuclear Medicine was established in Myanmar in 1963 by Dr Soe Myint and International Atomic Energy expert Dr R. Hochel at Yangon General Hospital. Nuclear medicine diagnostic and therapeutic services started with Probe Scintillation Detector Systems and rectilinear scanner. In the early stage, many Nuclear Medicine specialists from the International Atomic Energy Agency (IAEA) spent some time in Myanmar and made significant contributions to the development of Nuclear Medicine in our country. The department participated in various IAEA technical cooperation projects and regional cooperation projects. By the late 1990s, new centers were established in Mandalay, Naypyidaw, and North Okkalapa Teaching Hospital of University of Medicine 11, Yangon. The training program related to Nuclear Medicine includes a postgraduate master’s degree (three years) at the University of Medicine. Currently, all centers are equipped with SPECT, SPECT-CT, PET-CT, and cyclotron in Yangon General Hospital.

Up until now, the International Atomic Energy Agency has been playing a crucial role in the growth and development of Nuclear Medicine in Myanmar. Our vision is to provide a wide spectrum of nuclear medicine services at a level compatible with the international standards to become a Center of Excellence.

## Introduction

**Figure 1:**
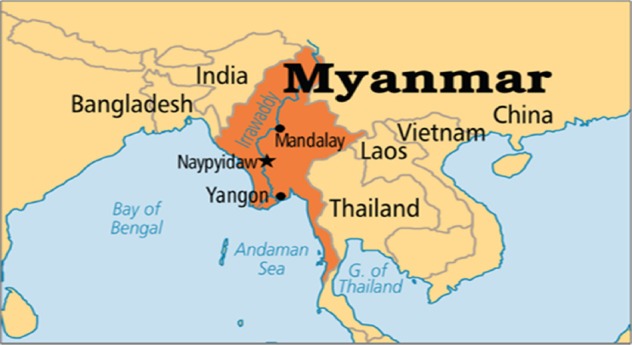
Map of Myanmar, which has area of 676,552 Sqkm (261,218 Sqmiles) and 52 million population

Myanmar (the Republic of the Union of Myanmar) located in South East Asia, has an area of 676,552 km^2^ (261,218 miles^2^) and a population of 52 million (1). Naypyidaw is the capital city, while Yangon is the largest city (Figure 1). Myanmar has a long and rich cultural history; the country was conquered by the British after three Anglo-Burmese Wars in the 19^th^ century, but it became an independent nation in 1948. Myanmar is rich in jade and gems, oil, natural gas, and other mineral resources.

Nuclear Medicine in Myanmar is an inde-pendent medical specialty operated by seven independent departments of nuclear medicine. The country in general and Nuclear Medicine in particular have benefited significantly from the support of the International Atomic Energy Agency (IAEA). IAEA has played and will continue to play a pivotal role not only in successful implementation of the projects, but also in sustainable growth and development of Myanmar Nuclear Medicine.

At present, Myanmar has a full range of nuclear medicine facilities including cyclotron and PET-CT. I have experienced the establishment and development of nuclear medicine in Myanmar throughout my lifetime. Thanks to the encouragement and introduction of Asian History of Nuclear Medicine (2, 3) by Prof. Henry Bom, the former president of the Asia Oceania Federation of Nuclear Medicine and Biology (AOFNMB) and the current chairman of the Asian Regional Cooperative Council for Nuclear Medicine (ARCCNM), I recalled my memories, collected data and photos, and described the history and perspectives of nuclear medicine in Myanmar.

### Beginning

**Figure 2:**
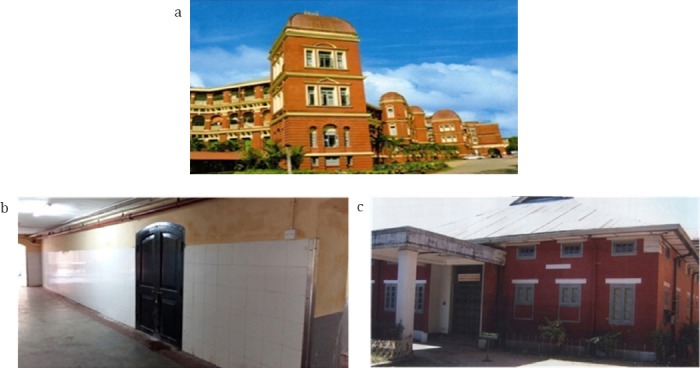
Yangon General Hospital, Department of Radioisotope, Department of Nuclear Medicine. a: Yangon General Hospital since 1899, b: Department of Radioisotpoe 1963-1966, c: Department of Nuclear Medicine 1966-

The Department of Radioisotope, the forerunner of the future Department of Nuclear Medicine, was founded in 1963 following the visits of the fact-finding mission in 1959, Dr E. H. Belcher, and the then Head of the Division of Life Sciences of IAEA and consultation with the local health authorities in the early 1963 (Figure 2). The first expertise service was received in June 1965. Thanks to relentless efforts and untiring endeavor of the co-founders, Dr R. Hoschl and Dr Soe Myint, the then Head of the Unit, a vast number of diagnostic tests were introduced over a four-year period including organ uptake studies, flow rate measurements, and basic hematological tests. Radioiodine therapy for thyrotoxicosis and thyroid cancer were also introduced (4).

### Step-up

**Figure 3:**
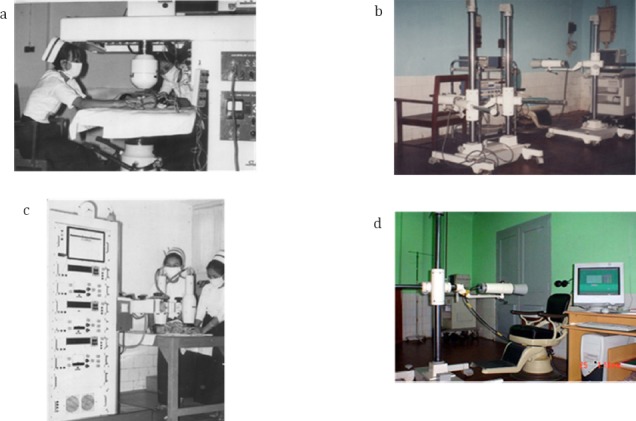
Rectilinear scanner and probe systems. a: 1966- IAEA BUR/6/04 Shimatzu Rectilinear Scanner b: 8.2.1985 IAEA BUR/6/014 Probe Counter, c: 1968- IAEA BUR/6/04 Probe Scintillation Detector, d: 2003 IAEA/RCA Thyroid Probe refurbish

With the receipt of the rectilinear scanner in 1966 and three probe scintillation detector systems in 1968 (Figure 3) under the IAEA Project BUR/6/04, the activities were further extended incorporating scintigraphy, renography, ^51^Cr-red blood cell survival, ferrokinetic studies with ^59^Fe, blood volume studies, and vitamin B12 absorption studies into the daily routines (4).

In 1974, the department was provided with a well counter with automatic sample changer, printer, and liquid scintillation counter under Project BUR/6/05. The second IAEA’s expert who took up assignment for six months in 1974 developed radiopharmacy techniques for preparation of generator-produced radiopha-rmaceuticals. With the successful development of radiopharmacy, in-house preparation of macroaggregated albumin (MAA) was used in lung scintigraphy (4).

The Japanese government donated Ohio Nuclear Gamma Camera in March 1975 on the request of the Department of Health, which started the use of gamma cameras in Myanmar. During that year, physicians, as well as in vivo and in vitro technologists, got IAEA fellowship grants and were sent to the UK to attend postgraduate studies. After their return to the department, placenta localization, ^32^P therapy in polycythemia vera, and ^198^Au colloidal gold for palliative treatment of the ascites caused by ovarian cancer were introduced.

**Figure 4:**
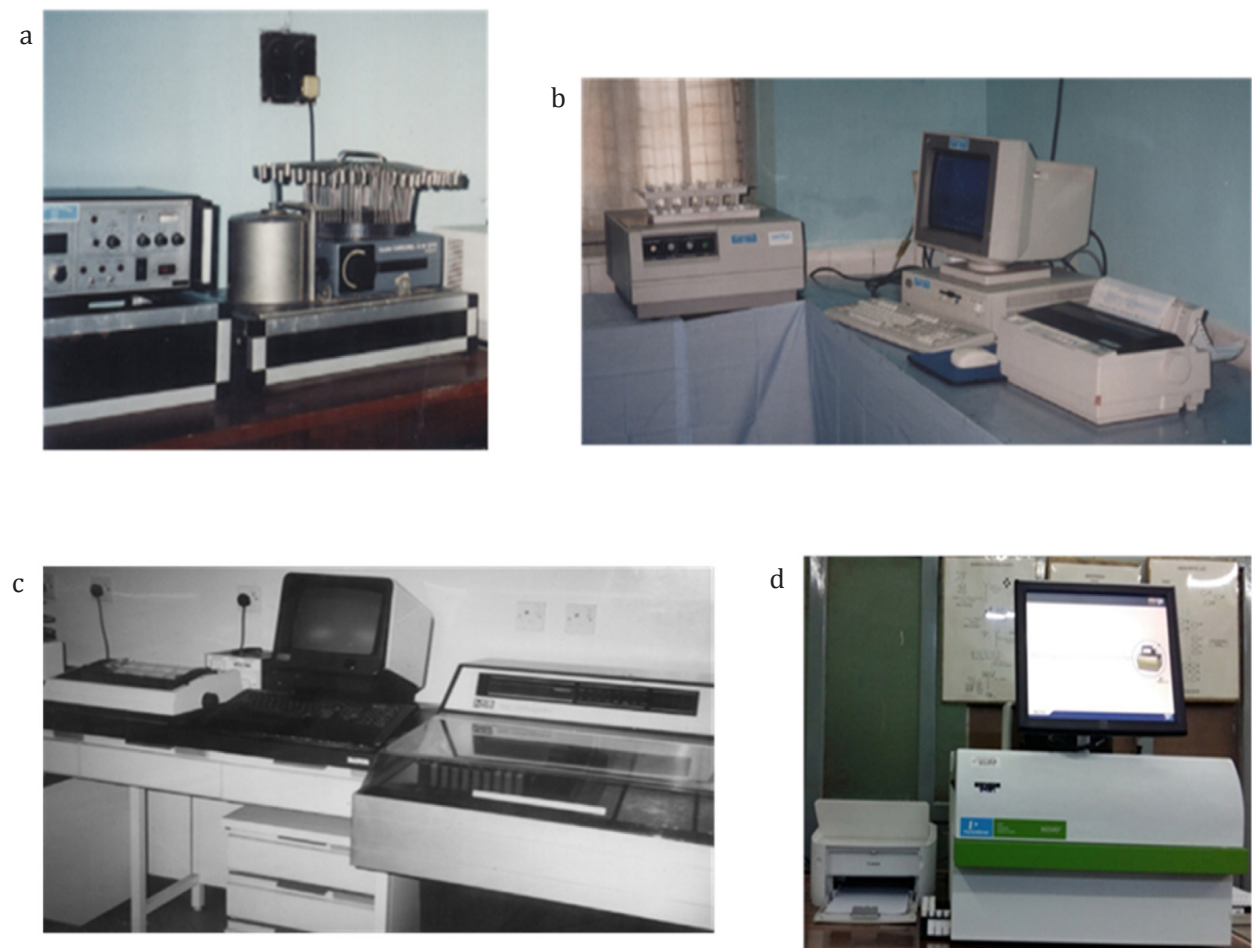
In vitro systems. a: 7.5.1985 IAEA Oakfield Gamma Counter, b: 3.3.1993 IAEA 12 Channel Sourcerer Gamma Counter, c: 18.2.1987 LKB Gamma Counter, d: 27.3.2014 Perkin Elmer Gamma Counter

The in vitro section of the department opened a new chapter in 1982 under the supervision of Dr R. D. Piyasena, who set up the radioimmunoassay procedures for thyroid-related, cortisol, and sex hormones using bulk reagents (4, 5). He also set up Nuclear Medicine in vitro at the Department of Medical Research in Yangon (Figure 4).

### New Departments, New Services

**Figure 5:**
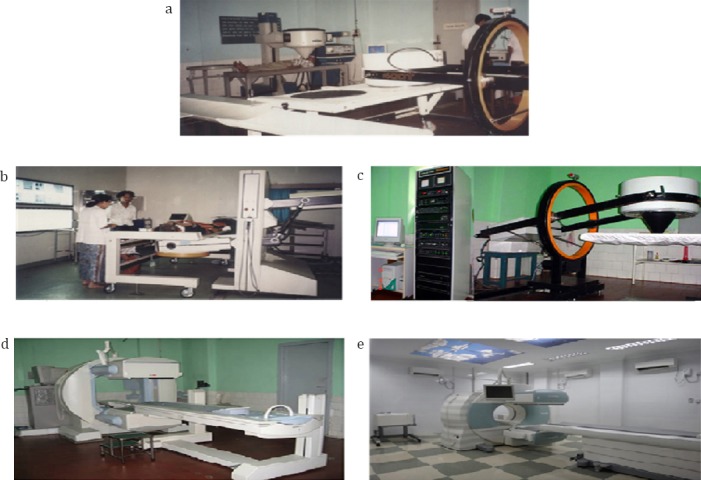
Gamma camera systems. a: 12.6.1985 Gamma muvek MB 9100 Gamma Camera, b: 18.2.1987 Hitachi Gamma Camera RC 135 DT, c: 24.5.1996 GE 400T Gamma Camera, d: 30.5.2003 SPECT Camera E Cam, e: 7.3.2014 SPECT CT Symbia

Japan International Cooperation Agency (JICA) provided a fully equipped Nuclear Medicine Unit at New General Yangon Hospital in February 1987 (Figure 5). In 1996, the diagnosis of hepatitis B infection was conducted (5). In March 1997, the silver jubilee of the Regional Cooperative Agreement, 19^th^ Meeting of the Working Group, was held in Yangon, Myanmar. In 2005, the department joined IAEA Distance Assisted Training for Nuclear Medicine Technologists. In 2006, the neonatal hypothyroid screening project was conducted in collaboration with the Department of Medical Research (4, 5).

In January 1998, the Ministry of Health inaugurated a new Department of Nuclear Medicine at Mandalay General Hospital, a teaching hospital affiliated to University of Medicine, Mandalay, the second largest city in Myanmar. In an attempt to provide extended diagnostic services, new departments were established at Thingangyun General Hospital and North Okkalapa Teaching Hospital of University of Medicine II of Yangon in 2002 and 2003, respectively.

In January 1999, a Postgraduate Diploma in Nuclear Medicine was inaugurated at the University of Medicine I in Yangon. After eight years, the one-year course was extended to a two-year master program in January 2007. In 2017, the course was extended to three years.

In 2009, in vivo and in vitro Diagnostic and Therapeutic Nuclear Medicine was established in a 1000 bedded specialist hospital at Naypyidaw, a new Administrative Capital of Myanmar. There is also a diagnostic Nuclear Medicine department In the 1000-bedded military hospital at Naypyidaw.

### Current Status and Perspectives

**Figure 6:**
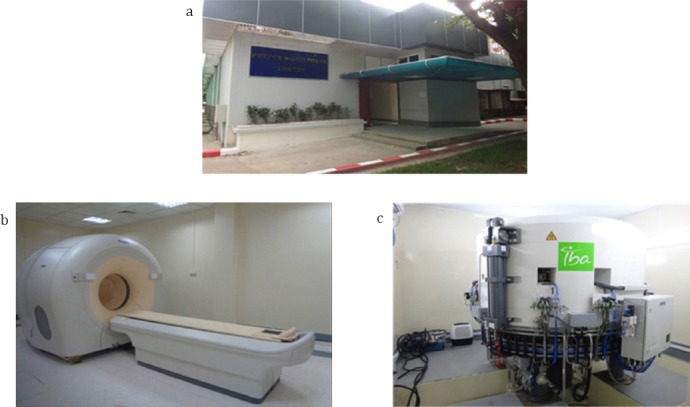
Positron emission tomography–computed tomography and cyclotron. a: PET CT and Cyclotron Center, b: 1.2.2015 PET CT Gemini, TOF,16 slices, c: 1.3.2016 Cyclotron 18 Mev Iba

At present, there are six nuclear medicine centers under the Ministry of Health and one in the private sector. Regarding the equipment, the first dual head SPECT camera was received in 2003, and Nuclear Cardiology imaging was established. In 2014, SPECT-CT cameras were installed in all centers (Figure 5). With the receipt of PET-CT in 2015 and cyclotron in 2016, Nuclear Oncology started to flourish (Figure 6). In the near future, more centers will be coming up in Shan States and Magwe Division, teaching hospitals of University of Medicine. In addition, PET-CT centers will emerge in private sector.

The department participated in various IAEA technical cooperation projects since 1963, IAEA regional cooperation agreement projects since 1987, and coordinated research projects (4, 5). Nuclear medicine professionals of Myanmar actively joined activities of the ARCCNM since 2003. The society maintains relationship with international organizations and acts as the host country in symposia, training courses, and meetings every year since 2012 (7). The society became a member state of the Asian School of Nuclear Medicine in May 2016 and member state of the World Federation of Nuclear Medicine and Biology in October 2016. The society successfully completed IAEA QUANUM audit in September 2016 (6).

Many experts in Nuclear Medicine from other countries have played major roles in the improvement of nuclear medicine services in Myanmar. These experts are Professor E. H. Belcher from Australia, Dr R. Hoschl from Czechoslovakia, Dr L. Kertez from Hungary, Dr R. D. Piyasena from Srilanka, Professor N. Kochupillar from India, Professor Sher M Khan and Dr Iftikhar from Pakistan, Dr Xie Yanfen from IAEA, Professor Felix X. Sundram from Malaysia, Dr Nandrani S. De Zoysa from Srilanka, Mr A. J. M. Bandula Seneveratna from Srilanka, Ms Veronica Wiley from Australia, Professor Brian Hutton from UK, Dr Sai Han from the UK, and Mr Simon Becher, Mr Chatri Hutakari, and Mr Robin Phoon from Thailand and Singapore GE Medical Co.

Our vision is to provide a wide spectrum of nuclear medicine services at a level compatible with internationally accepted standards and to become a Center of Excellence in providing efficient, updated, and timely nuclear medicine services.
